# Socioeconomic Status and Dental Care Utilization in Older Adults: A Comparison Between Australia and Japan

**DOI:** 10.1111/jphd.70035

**Published:** 2026-01-16

**Authors:** Urara Taguchi, My Tran, Sachiko Ono, Paul Kowal, Jun Aida, Kazuto Hoshi, Tomoko Sugiura

**Affiliations:** ^1^ Department of Oral Maxillofacial Surgery University of Tokyo Tokyo Japan; ^2^ National Centre for Health Workforce Studies, National Centre for Epidemiology and Population Health, College of Law, Governance and Policy Australian National University Canberra Australia; ^3^ Department of Eat‐Loss Medicine, Graduate School of Medicine The University of Tokyo Tokyo Japan; ^4^ Health Data Analytics Team, National Centre for Epidemiology and Population Health, Australian National University Canberra Australia; ^5^ Department of Dental Public Health, Graduate School of Medical and Dental Science Institute of Science Tokyo Tokyo Japan

**Keywords:** Australia, dental care for aged, dental care utilization, dental insurance, Japan, socioeconomic status

## Abstract

**Objectives:**

With the growing emphasis on incorporating dental care into universal health coverage (UHC) worldwide, it is essential to understand the extent to which UHC can improve access to needed health services without financial hardship. Dental care services should be included in monitoring progress toward UHC, but are often left out, even in countries with UHC. This study will compare socioeconomics‐related inequalities in dental care utilization among older Australians and Japanese, who experience contrasting universal dental insurance systems.

**Methods:**

We used data from Australia and Japan to estimate socioeconomics‐related inequalities in dental care utilization as the Slope Index of Inequality (SII) and the Relative Index of Inequality (RII) for community‐dwelling adults aged 65 years and older. Socioeconomic status was measured using equivalized income and educational attainment. Dental care utilization was defined as visiting a dentist within the past 12 months.

**Results:**

The study included 6104 Australian participants (mean age 73.8 years) and 19,043 Japanese participants (mean age 74.9 years). Income‐related inequalities in dental care utilization were higher in Australia [SII (0.22, 95% CI = 0.18–0.27); RII (1.48, 95% CI = 1.36–1.59)] than in Japan [SII (0.16, 95% CI = 0.14–0.19); RII (1.28, 95% CI = 1.24–1.33)]. We found a similar pattern in educational attainment.

**Conclusions:**

Notwithstanding the differences between the two dental care systems, the lack of UHC in dental care in Australia may be a contributing factor to greater inequalities in dental care utilization among older adults.

## 1 Introduction

Oral health is an essential aspect of health and well‐being, and significantly affects nutritional status, health, and quality of life over the life course [1, 2, 3]. Yet oral health and dental care have been largely absent from discussions about the United Nations Sustainable Development Goal 3.8, focusing on countries moving toward universal health coverage (UHC) [4]. UHC is intended to be a progressive expansion of health services coverage and financial protection, but even with increasing advocacy, little progress is being made on incorporating dental care into essential health services [5, 6, 7]. As populations age, the burden of chronic disease typically increases, including oral cavity chronic conditions such as tooth loss, dental infections, mucosal lesions, chewing, swallowing, and oral cancer. Many chronic systemic diseases and oral diseases share common risk factors, but poor oral health is not an inevitable consequence of aging, especially with access to dental care services that support good oral health over a lifetime [8]. So, with global population aging [9], addressing disparities in oral health and access to dental care services becomes more important.

Socioeconomic factors are known to influence both oral health and utilization of dental care among older adults [10, 11, 12, 13, 14]. Lower socioeconomic status individuals tend to have worse oral health and lower dental care utilization. Given the high price elasticity of demand for dental care, financial barriers are impactful [15, 16]. However, oral health care coverage for older adults of retirement age varies across countries, despite relatively small differences in their incomes [4]. While younger people and working‐age adults may receive dental check‐ups through school and workplace programs, older populations must seek care independently, often facing financial barriers that exacerbate disparities [17]. In addition, environmental factors such as community water fluoridation may help mitigate oral health inequalities. Australia has implemented community water fluoridation since 1953, whereas Japan has not. To date, few studies have compared countries with different insurance systems and their impact on oral health disparities. Because Japan has UHC that includes dental care, comparing dental care utilization between Japan and other countries without dental care coverage can be crucial to understanding the role of insurance systems. Only two previous studies have examined socioeconomic disparities in oral health between Japan and other countries (the United Kingdom and Singapore) [18, 19]. In the UK, dental care is provided under the National Health Service, with a banded system covering treatments from basic check‐ups to advanced procedures. In Singapore, most dental care is paid out‐of‐pocket, but certain treatments are partially covered through government‐mandated Medisave accounts (Supplementary Table A1). The previous studies used edentulism (complete tooth loss) as the outcome instead of dental care utilization and found smaller socioeconomic disparities in oral health in Japan compared with those in the United Kingdom and Singapore [18, 19]. However, while some previous studies have used edentulism as an outcome, no cross—country studies including Japan examining different health insurance systems and their impacts on dental care utilization have been published. More socioeconomically advantaged people may use more health services as they can afford them [20]. However, they also have better oral health, resulting in lower utilization of dental care services [21]. Thus, it is unclear what the mediating effect of dental care insurance is on dental care utilization. Comparing countries with different approaches to dental coverage can help to understand how health and social systems contribute to inequalities in dental care utilization [22] and provide evidence to support improving access to affordable dental care for older persons.

Examination of published research has not unearthed studies comparing socioeconomics‐related inequalities in dental care utilization between two countries with contrasting dental coverage systems. Japan and Australia serve as unique case studies to fill this gap in the existing literature. The two countries share several economic and geographical similarities (Supplementary Table A1). However, their health care systems differ, particularly in dental care coverage. Japan provides UHC, while Australia relies on private dental insurance. Thus, our study aims to compare socioeconomic inequities in dental care utilization, using the slope index of inequality (SII) [23] and relative index of inequality (RII) [23], among older persons in Australia and Japan, based on nationally representative data. Findings from this study will provide evidence to support access to affordable dental care for older people, thereby improving the older population's health, functioning, and well‐being.

## 2 Methods

### 2.1 Data Source and Study Population

Data from two nationally representative cross‐sectional cohort studies were used for the analyses: the 2021 Household, Income and Labor Dynamics in Australia (HILDA) Survey [24] and the 2022 Japan Gerontological Evaluation Study (JAGES) [25]. The HILDA survey included information on participants aged 15 years and older from each household, collecting data on various aspects of respondents' lives, including demographic characteristics, family dynamics, and health. The JAGES investigates social determinants of health among functionally independent adults aged 65 years and over in Japan. Both surveys include questions on the timing of the last dental visit, age, sex, and socioeconomic status.

The analytical sample included community‐dwelling participants aged 65 years and older who answered a question about their last dental visit in each study. The chosen analytical method was based on a complete case analysis, where invalid responses and missing data (HILDA: 4.9%, JAGES: 14.0%) were excluded. A missing pattern analysis was conducted to compare age, sex, and SES between excluded and included participants (Supplementary Table A2). As a robustness check, an analysis was done using imputed data. Overall, the findings were similar to the estimates using non‐imputed data. Thus, the estimates using the non‐imputed data were presented as the main results.

### 2.2 Health Care System

Japan and Australia differ substantially in their dental care coverage. Japan provides universal health insurance that includes a wide range of dental services, covering over 70% of costs through a copayment system (copayment rates: 30% for those < 70 years, 20% for 70–74, and 10% for ≥ 75). On the other hand, Australia's public health insurance system (Medicare) does not yet cover dental care services for adults. Access to dental care largely depends on private insurance, which is income‐tested.

### 2.3 Socioeconomic Status

Our variable of interest is socioeconomic status, measured using equivalized income and educational status. Equivalized income was calculated using the median value of the multiple‐choice annual household gross income ranges. First, the Australian Dollar (AUD) and Japanese Yen (JPY) values were adjusted using purchasing power parity (PPP) to align both currencies to the 2021 US Dollar (USD) (1USD (2021) = 1.41AUD (2021), 1USD (2021) = 101.59JPY (2022)) [26]. As the income data were collected at different times for both countries, the PPP adjustment considers not only exchange rate differences between countries but also inflation changes within each country over time. Second, gross household income was converted to equivalized income to account for differences in household size and composition, based on previous studies (see Appendix A1) [27, 28]. Third, equivalized income was divided into quartiles based on its income ranges. This allowed for comparison of Australians and Japanese of the same socioeconomic positions within their countries. Educational status was categorized into two groups: One for individuals with 12 or fewer years of education and another for those with more than 12 years of completed education. This classification accounts for the differences in the education systems between Australia and Japan (see Appendix A2).

### 2.4 Dental Care Utilization

A question on time since the last dental visit was used to create the outcome variable, which equals one if the person reported visiting a dentist within the past 12 months, and zero otherwise. Neither study asked about more than one visit, but this categorization aligns with clinical guidelines that define “regular attendance” as at least one visit every 6–12 months [29].

### 2.5 Covariates

Covariates were selected based on previous studies and consistency between the two datasets, including age (continuous) and sex (males and females) [18].

### 2.6 Statistical Analysis

To examine both absolute and relative inequalities in dental care utilization between Australia and Japan, the SII and the RII were estimated, unadjusted and adjusted by age and sex using logistic regression models [23, 30]. SII quantifies the absolute difference in health outcome across socioeconomic groups, considering the full distribution of socioeconomic status rather than only the extremes. The value of SII = 0 indicates equality, and higher values represent increasing inequality. RII, on the other hand, expresses the relative difference in health outcomes across socioeconomic groups. The value of RII = 1 indicates equality, and higher values represent increasing inequality. A sampling weight was applied in the HILDA data analysis to ensure population representativeness, whereas no sampling weights were used for the JAGES data due to the limitation of its study design.

Prevalence ratios were generated using the robust Poisson regression method adjusted for age and sex. This method fits a Poisson regression model using a log scale for the outcome and applies robust standard errors [31].

For sensitivity analysis, an analysis using imputed data was done to assess potential non‐random missing bias. Missing values for the exposure variables (income and educational status) were generated using multiple imputations by chained equations, with estimates pooled across 20 datasets. To ensure comparability between the two countries, the complete‐case data were analyzed using Australian equivalized income recalculated using the same method used in the Japanese dataset, which is based on the OECD formula. For heterogeneity analysis, SII and RII were estimated on different Australian sub‐samples, based on the presence of private dental insurance and pensioner status, to compare the inequalities between insured Japanese and Australians. To examine the heterogeneity in inequalities in disadvantaged communities, the SII and RII were generated using the subsamples of urban and non‐urban areas (see Appendix A3) [32, 33]. Lastly, the Japanese data were stratified by age groups corresponding to different copayment rates (< 70 years, 70–74 years, ≥ 75 years). All statistical analyses were performed using R (version 4.2.0; R Foundation for Statistical Computing, Vienna, Austria).

## 3 Results

### 3.1 Participants

**FIGURE 1:**
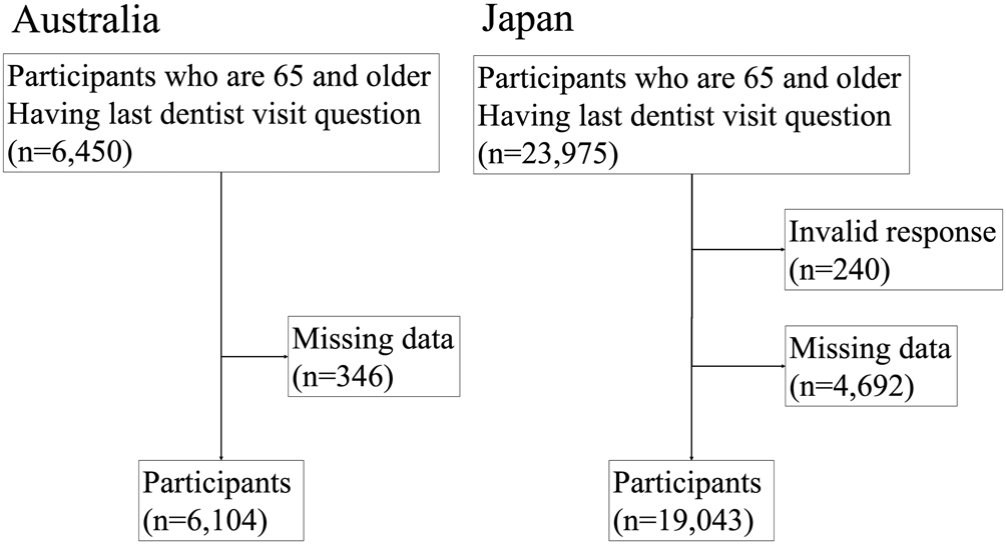
Flowchart of participant selection.

Figure 1 shows a flowchart of the participants' selection for both Australia and Japan. In the Australian dataset, from a total sample of 16,549, 6450 individuals responded to the question regarding their last dental visit in 2021. Of these, 346 were excluded due to missing data, leaving 6104 respondents. In the Japanese dataset, from a total sample of 338,742, 23,975 individuals responded to the corresponding question on dental visits. Among them, 240 were excluded due to invalid responses and 4692 due to missing data. As a result, 19,043 respondents were included in the final analysis.

### 3.2 Summary Statistics

The proportion of individuals aged 75 years or older was higher in Japan than in Australia (see Table 1). In contrast, Australia had a higher proportion of respondents with more than 12 years of education. The HILDA sample also includes a higher proportion of females and a larger non‐urban population than JAGES. The characteristics of those with missing data are shown in Supplementary Table A3. The proportion of missing data for equivalized income was 4.9% in HILDA and 14% in JAGES. For educational status, the missing rates were 0.3% in HILDA and 2.1% in JAGES.

**TABLE 1:** Characteristics of participants in HILDA and JAGES.

		Australia (*n* = 6104)	Japan (*n* = 19,043)
Age, years (mean (SD))		73.8 (6.8)	74.9 (6.4)
Age	65–74 years	3712 (60.8)	10,074 (52.9)
	≥ 75 years	2392 (39.2)	8969 (47.1)
Sex	Male	2946 (48.3)	9570 (50.3)
	Female	3158 (51.7)	9473 (49.7)
Educational status	≥ 13 years	3382 (55.4)	6704 (35.2)
	≤ 12 years	2722 (44.6)	12,339 (64.8)
Equivalized income	Very low	1910 (31.3)	4630 (24.3)
	Low	1521 (24.9)	4797 (25.2)
	Medium	1439 (23.6)	4902 (25.7)
	High	1234 (20.2)	4714 (24.8)
Last dental visit	≥ 13 months	2545 (41.7)	6084 (31.9)
	≤ 12 months	3559 (58.3)	12,959 (66.5)
Place of residence	Non‐urban	2718 (44.5)	5199 (26.7)
	Urban	3386 (55.5)	14,278 (73.3)

*Note:* Data are presented as *n* (%) unless otherwise indicated.

**FIGURE 2:**
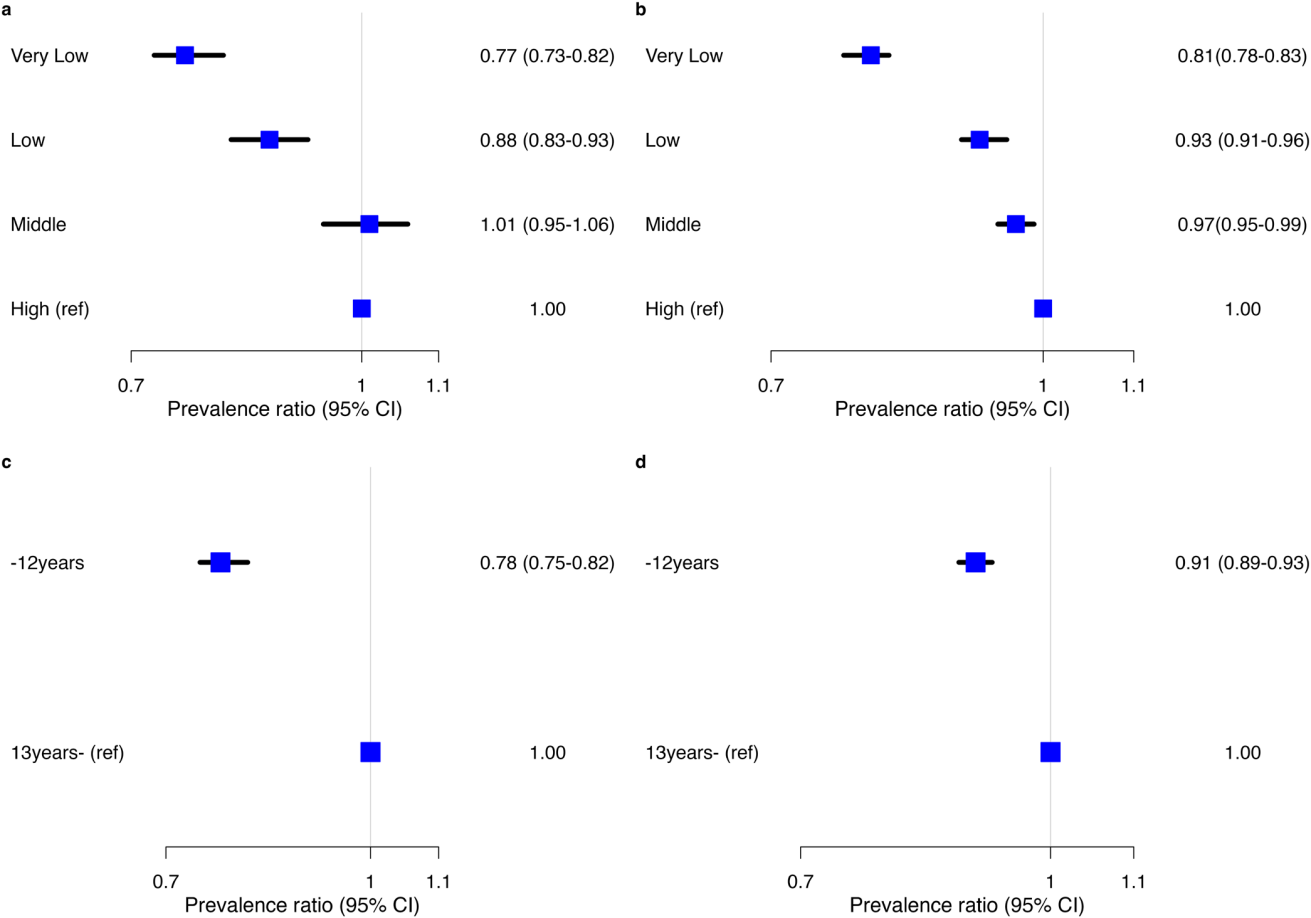
Prevalence ratio of dental care utilization within 12 months by income status and educational status. (a) Prevalence ratio for income status in Australia. (b) Prevalence ratio for income status in Japan. (c) Prevalence ratio for educational status in Australia. (d) Prevalence ratio for educational status in Japan, 95% CI: 95% confidence interval. [Color figure can be viewed at wileyonlinelibrary.com]

Figure 2 shows the age and sex adjusted association between socioeconomic status and last dental visit. In both countries, lower socioeconomic status was associated with lower dental care utilization.

### 3.3 Main Findings

Table 2 presents the SII and RII for dental care utilization in Australia and Japan. Regarding equivalized income status, both SII and RII, adjusted for age and sex, were higher in Australia than in Japan. A similar pattern was observed for educational attainment in Australia.

**TABLE 2:** Estimated SIIs and RIIs in Australia and Japan.

		Model	Australia		Japan	
			Estimate	95% CI	Estimate	95% CI
SII	Income status	Unadjusted	0.22	0.18–0.27	0.16	0.14–0.19
		Adjusted	0.22	0.18–0.27	0.16	0.14–0.19
	Educational status	Unadjusted	0.25	0.21–0.30	0.12	0.10–0.15
		Adjusted	0.25	0.21–0.30	0.12	0.10–0.15
RII	Income status	Unadjusted	1.48	1.36–1.60	1.28	1.24–1.33
		Adjusted	1.48	1.36–1.59	1.28	1.24–1.33
	Educational status	Unadjusted	1.56	1.42–1.70	1.21	1.15–1.26
		Adjusted	1.56	1.42–1.70	1.21	1.15–1.26

*Note:* The adjusted model included age and sex.

Abbreviations: CI = confidence interval, RII = relative index of inequality, SII = slope index of inequality.

These findings indicate that dental care utilization was concentrated among those with higher socioeconomic status, using both income and educational attainment. However, socioeconomic‐related inequities in dental care utilization were found to be larger in Australia (with no public dental coverage) than in Japan (with public dental coverage).

### 3.4 Sensitivity and Heterogeneity Analysis

The SII and RII using imputed data and the OECD formula for equivalized income were similar to the main analysis (see Supplementary Table A4).

Regarding subgroup analysis, inequalities were significantly lower among Australians with dental insurance (Supplementary Table A5), suggesting that insurance coverage could reduce the disparities in dental care utilization. However, since the cost of Australian dental insurance is mainly out‐of‐pocket, such effects would be highly concentrated in the above‐middle‐income group. The SII was similar across urban and non‐urban areas, whereas the RII was higher in non‐urban areas (Supplementary Table A6). In Japan, the income‐related RII in non‐urban areas was higher than that found in Australia. SII and RII were similar across age groups in Japan. Co‐payment levels may not have an impact on inequalities (Supplementary Table A7).

## 4 Discussion

This study compared the socioeconomic‐related inequalities in dental care utilization among older adults in Australia and Japan. Older adults in Australia had higher levels of inequality in dental care utilization based on income and educational status compared to those in Japan.

Previous studies have shown that socioeconomic inequalities in edentulism among older adults are smaller in Japan than in other countries [18, 19]. Although some differences in magnitude were observed, the findings in this study are consistent with those in previous studies; greater inequality in dental care utilization was observed in Australia compared to Japan. The differences in SII and RII between Singapore and Japan in the previous study were greater than those between Australia and Japan in the current study. This can be attributed to a smaller non‐urban area and the lower dentist‐to‐population ratio in Singapore. In contrast, Japan and Australia share similar characteristics in terms of geography and dentist‐to‐population ratios (Supplementary Table A1), allowing a clearer assessment of the impact of insurance system differences. By focusing on actual utilization behavior, this study provides a more practical and policy‐relevant approach, allowing for assessment of structural factors such as public dental care coverage and the geographic distribution of dentists. Furthermore, our analyses accounting for residence in non‐urban areas, private dental care coverage, and reliance on pension income revealed additional layers of inequality.

The socioeconomic‐related inequalities in dental care utilization found in this study may be partly explained by several possible mechanisms between the two countries. A previous study showed that reduced out‐of‐pocket costs through public health care coverage were associated with improved access to health services, including dental care, which has been recognized as having high price elasticity of demand [34]. Low‐income populations are more likely to receive needed dental services when care is provided free of charge. In countries where out‐of‐pocket dental expenses are high, those with lower income or no private insurance are less likely to seek care, leading to greater disparities. In addition to insurance coverage, contextual factors such as distribution of resources, including the place of residence [35], and availability of dental professionals [36], may also contribute to inequality in dental care utilization. Previous studies have shown that older adults living in areas with a higher dentist‐to‐population ratio and in urban areas are more likely to utilize dental services. In Australia, the dentist‐to‐population ratio is lower than in Japan, and the non‐urban population is larger. These factors may help explain why socioeconomic‐related inequalities in dental care utilization were larger in Australia compared with Japan in this study. Given the growing aging population, our results support the consideration of expanding public dental care coverage as a potential policy to reduce socioeconomic disparities in dental care utilization.

There are several limitations in this study that need to be acknowledged. First, the oral health status was unknown due to the unavailability of information. Second, wealth‐related inequalities, a measure of lifetime income in the older population, were not assessed due to the lack of wealth information in the datasets. Older adults often possess more assets, even if they do not have a regular source of income after the age of 65. Previous studies have shown that higher wealth is associated with more frequent dental care utilization [14, 37]. Third, the HILDA dataset included community‐dwelling older adults, some of whom may require assistance, whereas the JAGES dataset included only independent individuals, which may have led to an underestimation of dental care utilization in Australia. Fourth, since JAGES data were collected in collaboration with partner municipalities, which were not randomly selected, the sample may not be fully nationally representative. Unfortunately, sampling weights were not available for JAGES, which could potentially impact the generalizability of our findings. Fifth, selection and recall biases could exist in the self‐reported data. Survey respondents tend to be more independent and have higher levels of education, which may introduce selection bias [38]. As for recall bias, this bias may lead to both under‐ and overestimation of the inequalities [38]. However, these potential biases are unlikely to have a significant impact on this study since this effect is likely to be similar in both countries. Sixth, there are differences in community water fluoridation between the two countries. Although community water fluoridation is most effective for children aged 5–8 years [39], it was only partially implemented in Australia starting in 1953; thus, few of those aged 65 and older in 2021 would have fully benefited. As our study focuses on dental care utilization, the impact of historical community water fluoridation exposure is likely minimal. Finally, causal relationships could not be assessed in this study. Further research would help to better understand the impact of public insurance coverage on dental care utilization and oral health in both countries.

## 5 Conclusions

By evaluating socioeconomic‐related inequalities in dental care utilization among older adults between Australia and Japan, based on the presence or absence of public dental care coverage, this study reveals disparities in dental care utilization and provides the role of public dental care coverage as a potentially mediating factor in reducing such disparities. These findings could contribute to the development of future dental care policies.

## Funding

This study was supported by JST SPRING (grant number JPMJSP2108).

This work was supported by the Japan Society for the Promotion of Science (JSPS) KAKENHI (Grant Numbers: 20H00557, 20K10540, 21K19635, 21H03196, 21K17302, 22H00934, 22H03299, 22K04450, 22K13558, 22K17409, 23K27807, 23H00449, 23H03117, 23K21500), Health Labor Sciences Research Grants (Grant Numbers: 19FA1012, 19FA2001, 21FA1012, 22FA2001, 22FA1010, 22FG2001), the Research Institute of Science and Technology for Society (RISTEX, Grant Number: JPMJOP1831) from the Japan Science and Technology Agency (JST), a grant from the Japan Health Promotion & Fitness Foundation, a TMDU priority research areas grant, and the National Research Institute for Earth Science and Disaster Resilience. This study used data from the Japan Gerontological Evaluation Study (JAGES). The views expressed in this paper are those of the authors and do not necessarily reflect the views of the funding agencies.

## Ethics Statement

The HILDA study was granted ethical approval by the Office of Research Ethics and Integrity at the University of Melbourne (reference No. 27248‐43,354‐3).

The JAGES study received approval from the ethics committee of the National Center for Geriatrics and Gerontology (No. 992) and Chiba University (No. 2493).

This study used anonymized secondary data sources for analyses. The data included no personal identifiers.

## Conflicts of Interest

The authors declare no conflicts of interest.

## Supporting information


**Data S1:** Supporting Information.

## Data Availability

The data used in this study are hosted by the data custodian: Melbourne Institute of Applied Economic and Social Research, and the JAGES project team. Due to agreements with the data custodians, the data are not to be shared publicly. Request for the data can be made via the Australia Data Archive: https://melbourneinstitute.unimelb.edu.au/hilda/for‐data‐users, and the JAGES data custodians: https://www.jages.net/kenkyuseika/datariyou/.
