# Supplementary material for: Socioeconomic Status and Dental Care Utilization in Older Adults: A Comparison Between Australia and Japan
Source: J Public Health Dent. 2026 Jan 16;86(1):81–7. doi: 10.1111/jphd.70035 (PMC12972269; doi:10.1111/jphd.70035)
Supplement: Supplementary file 1 — Data S1: Supporting Information. [file JPHD-86-81-s001.docx]

**Supplementary material**

**Appendix A1. Calculation of equivalized income**

The Australian Bureau of Statistics’ formula, as follow: $Equivalized income=\frac{\boldsymbol{Median value of income range}}{1+0.5\times\left( No. of adults-1 \right)+0.3\times(No. of children)}$

The OECD formula, as follows$: Equivalized income=\frac{Median value of income range}{\sqrt{\boldsymbol{number of people living together}}}$

**Appendix A2. Educational system in Australia and Japan**

In Australia the compulsory education lasts until Year 10 in most states (although in some states, Year 1 starts earlier), followed by optional senior secondary education (year 11 - 12), vocational training, or university. In Japan, compulsive education consists of six years elementary school and three years of junior high school (a total of nine years). High school lasts three years, followed by higher education such as vocational training or university.

**Appendix A3. Urban-rural typology**

In Australia, urban areas were defined as “major cities” based on the accessibility/remoteness index of Australia (ARIA+). The OECD urban-rural typology was applied in Japan, which defines rural areas as those with a population density below 500 inhabitants per km^2^.

**Supplementary Table A1**. **Similarity and difference between Australia, Japan, UK and Singapore.**

|  | **Australia** | **Japan** | **UK** | **Singapore** |
| --- | --- | --- | --- | --- |
| **Region** | Asia-Pacific | Asia-Pacific | Western | Asia-Pacific |
| **Income Group** | High-income (OECD member) | High-income (OECD member) | High-income (OECD member) | High-income (non-OECD member) |
| **Geography** | Surrounded by seas | Surrounded by seas | Surrounded by seas | Connected to the mainland (Malaysia) |
|  | Having remote area | Having remote area | Not having remote area | Not having remote area |
|  | An extensive territory | Longitudinally extended | Small country | Small country |
| **Dentists per population** | 6.41 per 10,000 people | 8.16 per 10,000 people | 4.93 per 10,000 people | 4.61 per 10,000 people |
| **Universal care coverage index** | 87 (2021) | 83 (2021) | 88 (2021) | 89 (2021) |
| **Water fluoridation** | 1953 - | None | 1964 - | 1956 - |
| **Medical care coverage** | Covered by public health insurance (Medicare) | Covered by public health insurance (National health insurance) | Covered by public health insurance (National Health Service) | Covered by public medical saving account (Medisave) |
| **Dental care coverage** | Not covered by public health insurance | Covered by public health insurance | Covered by public health insurance | Mostly out-of-pocket, some via Medisave |

**Supplementary Table A2. Missing pattern of participants.**

|  |  | **Australia** | |  | **Japan** | |
| --- | --- | --- | --- | --- | --- | --- |
|  |  | **Not missing** | **Missing** |  | **Not missing** | **Missing** |
| **Age (mean (SD))** |  | 73.9 (6.8) | 75.4 (4.5) |  | 75.2 (6.5) | 77.9 (7.1) |
| **Sex (n (%))** | **Male** | 3,346 (99.6) | 12 (0.4) |  | 11,869 (96.1) | 487 (3.9) |
|  | **Female** | 3,088 (99.9) | 4 (0.1) |  | 10,764 (96.3) | 415 (3.7) |
| **Equivalized income (n (%))** | **Very low** | 1,912 (99.6) | 8 (0.4) |  | 4,911 (95.1) | 253 (4.9) |
|  | **Low** | 1,526 (99.7) | 2 (0.1) |  | 4,978 (97.1) | 147 (2.9) |
|  | **Medium** | 1,449 (99.7) | 5 (0.3) |  | 5,060 (97.8) | 113 (2.2) |
|  | **High** | 1,235 (100.0) | 0 (0.0) |  | 4,850 (98.1) | 95 (1.9) |
| **Educational status (n (%))** | **≥ 13 years** | 2,886 (99.8) | 6 (0.2) |  | 1,4801 (96.2) | 583 (3.8) |
|  | **≤ 12 years** | 3,528 (99.7) | 10 (0.3) |  | 7,629 (97.3) | 212 (2.7) |

**Supplementary Table A3. Characteristics of participants with missing data.**

|  |  |  | **Australia** |  | **Missing** |  | **Japan** |  | **Missing** |
| --- | --- | --- | --- | --- | --- | --- | --- | --- | --- |
|  |  |  | **N** | **%** | **%** |  | **N** | **%** | **%** |
| **Age** | **65–74 years** |  | 3,900 | 60.5 | 0.0 |  | 12,078 | 50.1 | 0.8 |
|  | **≥ 75 years** |  | 2,550 | 39.5 |  |  | 11,657 | 49.5 |  |
| **Sex** | **Male** |  | 3,092 | 47.9 | 0.0 |  | 11,179 | 47.5 | 0.8 |
|  | **Female** |  | 3,358 | 52.1 |  |  | 12,556 | 52.9 |  |
| **Educational status** | **≥ 13 years** |  | 3,538 | 55.0 | 0.3 |  | 7,841 | 33.8 | 2.1 |
|  | **≤ 12 years** |  | 2,912 | 45.1 |  |  | 15,894 | 67.0 |  |
| **Equivalized income** | **Very low** |  | 1,920 | 31.3 | 4.9 |  | 5,164 | 25.3 | 14 |
|  | **Low** |  | 1,528 | 24.9 |  |  | 5,125 | 25.1 |  |
|  | **Medium** |  | 1,454 | 23.7 |  |  | 5,173 | 25.3 |  |
|  | **High** |  | 1,235 | 20.2 |  |  | 4,945 | 24.2 |  |

**Supplementary Table A4. Prevalence ratios, SSI and RII – Sensitivity checks.**

|  |  |  | **Australia** | |  | **Australia** | |  | **Japan** | |
| --- | --- | --- | --- | --- | --- | --- | --- | --- | --- | --- |
|  |  |  | **Imputed data** | |  | **Recalculated equivalized income** | |  | **Imputed data** | |
|  |  |  | **Estimate** | **95% CI** |  | **Estimate** | **95% CI** |  | **Estimate** | **95% CI** |
| **Prevalence ratio** | **Income** | **Very low** | 0.78 | 0.71–0.85 |  | 0.78 | 0.73–0.83 |  | 0.80 | 0.76–0.84 |
|  |  | **Low** | 0.88 | 0.80–0.97 |  | 0.88 | 0.83–0.94 |  | 0.93 | 0.89–0.97 |
|  |  | **Middle** | 1.00 | 0.92–1.10 |  | 1.01 | 0.95–1.07 |  | 0.97 | 0.92–1.01 |
|  |  | **High** | Ref. | Ref. |  | Ref. | Ref. |  | Ref. | Ref. |
|  | **Education** | **≤ 12 years** | 0.79 | 0.74–0.85 |  | - | - |  | 0.90 | 0.87–0.94 |
|  |  | **≥ 13 years** | Ref. | Ref. |  | - | - |  | Ref. | Ref. |
| **SII** | **Income** | **Unadjusted** | 0.20 | 0.16–0.25 |  | 0.22 | 0.18–0.26 |  | 0.06 | 0.04–0.09 |
|  |  | **Adjusted** | 0.20 | 0.16–0.25 |  | 0.22 | 0.18–0.26 |  | 0.06 | 0.04–0.09 |
|  | **Education** | **Unadjusted** | 0.23 | 0.18–0.27 |  | - | - |  | 0.07 | 0.04–0.10 |
|  |  | **Adjusted** | 0.23 | 0.18–0.27 |  | - | - |  | 0.07 | 0.04–0.10 |
| **RII** | **Income** | **Unadjusted** | 1.43 | 1.31–1.54 |  | 1.47 | 1.35–1.59 |  | 1.10 | 1.06–1.14 |
|  |  | **Adjusted** | 1.43 | 1.32–1.54 |  | 1.47 | 1.35–1.58 |  | 1.10 | 1.06–1.14 |
|  | **Education** | **Unadjusted** | 1.43 | 1.32–1.54 |  | - | - |  | 1.10 | 1.05–1.16 |
|  |  | **Adjusted** | 1.43 | 1.32–1.54 |  | - | - |  | 1.10 | 1.05–1.16 |

**Supplementary Table A5. Prevalence ratio, SII, and RII stratified by private insurance status in Australia.**

|  |  |  | **With private insurance** | |  | **Without private insurance** | |  | **Without private insurance & pensioners** | |  | **Without private insurance & non-pensioners** | |
| --- | --- | --- | --- | --- | --- | --- | --- | --- | --- | --- | --- | --- | --- |
|  |  |  | **Estimate** | **95% CI** |  | **Estimate** | **95% CI** |  | **Estimate** | **95% CI** |  | **Estimate** | **95% CI** |
| **Prevalence ratio** | **Income** | **Very low** | 0.89 | 0.83–0.95 |  | 1.02 | 0.85–1.22 |  | 1.40 | 1.01–1.94 |  | 0.98 | 0.75–1.27 |
|  |  | **Low** | 1.05 | 0.99–1.11 |  | 0.85 | 0.70–1.03 |  | 1.13 | 0.80–1.59 |  | 0.94 | 0.70–1.27 |
|  |  | **Middle** | 1.06 | 1.00–1.12 |  | 1.11 | 0.91–1.34 |  | 1.39 | 0.98–1.98 |  | 1.07 | 0.85–1.34 |
|  |  | **High** | Ref. | Ref. |  | Ref. | Ref. |  | Ref. | Ref. |  | Ref. | Ref. |
|  | **Education** | **≤ 12 years** | 0.89 | 0.85–0.93 |  | 0.80 | 0.72–0.89 |  | 0.83 | 0.73–0.93 |  | 0.84 | 0.69–1.02 |
|  |  | **≥ 13 years** | Ref. | Ref. |  | Ref. | Ref. |  | Ref. | Ref. |  | Ref. | Ref. |
| **SII** | **Income** | **Unadjusted** | 0.09 | 0.03–0.14 |  | 0.0028 | -0.06–0.06 |  | -0.09 | -0.008 – -0.17 |  | 0.01 | -0.15–0.18 |
|  |  | **Adjusted** | 0.09 | 0.03–0.14 |  | 0.0026 | -0.07–0.076 |  | -0.09 | -0.007 – -0.17 |  | 0.03 | -0.13–0.20 |
|  | **Education** | **Unadjusted** | 0.09 | 0.03–0.14 |  | 0.0012 | -0.11–0.12 |  | 0.11 | 0.03–0.20 |  | 0.15 | -0.03–0.34 |
|  |  | **Adjusted** | 0.09 | 0.03–0.14 |  | 0.0071 | -0.11–0.12 |  | 0.11 | 0.02–0.20 |  | 0.13 | -0.04–0.30 |
| **RII** | **Income** | **Unadjusted** | 1.13 | 1.05–1.21 |  | 0.09 | 0.82–1.19 |  | 0.78 | 0.61–0.96 |  | 1.03 | 0.70–1.36 |
|  |  | **Adjusted** | 1.13 | 1.05–1.22 |  | 1.01 | 0.82–1.20 |  | 0.78 | 0.61–0.96 |  | 1.07 | 0.72–1.41 |
|  | **Education** | **Unadjusted** | 1.13 | 1.05–1.21 |  | 1.00 | 0.71–1.30 |  | 1.36 | 1.04–1.69 |  | 1.35 | 0.85–1.85 |
|  |  | **Adjusted** | 1.13 | 1.05–1.22 |  | 1.02 | 0.72–1.31 |  | 1.36 | 1.03–1.68 |  | 1.30 | 0.84–1.76 |

**Supplementary Table A6. Prevalence ratio, SII, and RII - Urban vs non-urban areas in Australia and Japan.**

|  |  |  | **Australia – urban** | |  | **Japan – urban** | |  | **Australia – non-urban** | |  | **Japan – non-urban** | |
| --- | --- | --- | --- | --- | --- | --- | --- | --- | --- | --- | --- | --- | --- |
|  |  |  | **Estimate** | **95% CI** |  | **Estimate** | **95% CI** |  | **Estimate** | **95% CI** |  | **Estimate** | **95% CI** |
| **Prevalence ratio** | **Income** | **Very low** | 0.88 | 0.81–0.95 |  | 0.83 | 0.80–0.86 |  | 0.68 | 0.61–0.75 |  | 0.82 | 0.76–0.87 |
|  |  | **Low** | 0.94 | 0.88–1.02 |  | 0.93 | 0.91–0.96 |  | 0.80 | 0.72–0.89 |  | 0.96 | 0.90–1.03 |
|  |  | **Middle** | 1.01 | 0.94–1.09 |  | 0.97 | 0.95–1.00 |  | 1.00 | 0.92–1.10 |  | 0.98 | 0.92–1.05 |
|  |  | **High** | Ref. | Ref. |  | Ref. | Ref. |  | Ref. | Ref. |  | Ref. | Ref. |
|  | **Education** | **≤ 12 years** | 0.80 | 0.76–0.85 |  | 0.92 | 0.90–0.94 |  | 0.78 | 0.72–0.84 |  | 0.94 | 0.89–0.98 |
|  |  | **≥ 13 years** | Ref. | Ref. |  | Ref. | Ref. |  | Ref. | Ref. |  | Ref. | Ref. |
| **SII** | **Income** | **Unadjusted** | 0.13 | 0.07–0.19 |  | 0.15 | 0.12–0.17 |  | 0.10 | 0.03–0.17 |  | 0.16 | 0.11–0.21 |
|  |  | **Adjusted** | 0.13 | 0.07–0.19 |  | 0.15 | 0.12–0.17 |  | 0.10 | 0.04–0.17 |  | 0.16 | 0.11–0.21 |
|  | **Education** | **Unadjusted** | 0.15 | 0.08–0.21 |  | 0.10 | 0.07–0.13 |  | 0.10 | 0.03–0.17 |  | 0.10 | 0.04–0.16 |
|  |  | **Adjusted** | 0.15 | 0.08–0.21 |  | 0.10 | 0.07–0.13 |  | 0.10 | 0.04–0.17 |  | 0.10 | 0.04–0.16 |
| **RII** | **Income** | **Unadjusted** | 1.04 | 0.95–1.14 |  | 1.23 | 1.19–1.28 |  | 1.21 | 1.05–1.36 |  | 1.32 | 1.21–1.44 |
|  |  | **Adjusted** | 1.04 | 0.95–1.14 |  | 1.23 | 1.19–1.28 |  | 1.21 | 1.06–1.36 |  | 1.32 | 1.21–1.44 |
|  | **Education** | **Unadjusted** | 1.04 | 0.95–1.14 |  | 1.16 | 1.11–1.21 |  | 1.21 | 1.05–1.36 |  | 1.18 | 1.06–1.31 |
|  |  | **Adjusted** | 1.04 | 0.95–1.14 |  | 1.16 | 1.11–1.21 |  | 1.21 | 1.06–1.36 |  | 1.18 | 1.06–1.31 |

**Supplementary Table A7. Prevalence ratio, SII, and RII stratified by age groups in Japan.**

|  |  |  | **Japan – Under 69 years old** | |  | **Japan – 70-74 years old** | |  | **Japan – Over 75 years old** | |
| --- | --- | --- | --- | --- | --- | --- | --- | --- | --- | --- |
|  |  |  | **Estimate** | **95% CI** |  | **Estimate** | **95% CI** |  | **Estimate** | **95% CI** |
| **Prevalence ratio** | **Income** | **Very low** | 0.80 | 0.75–0.86 |  | 0.79 | 0.75–0.84 |  | 0.81 | 0.78–0.85 |
|  |  | **Low** | 0.90 | 0.85–0.96 |  | 0.90 | 0.86–0.95 |  | 0.95 | 0.91–0.98 |
|  |  | **Middle** | 0.97 | 0.92–1.02 |  | 0.95 | 0.91–1.00 |  | 0.97 | 0.94–1.01 |
|  |  | **High** | Ref. | Ref. |  | Ref. | Ref. |  | Ref. | Ref. |
|  | **Education** | **≤ 12 years** | 0.89 | 0.86–0.93 |  | 0.92 | 0.89–0.95 |  | 0.91 | 0.88–0.93 |
|  |  | **≥ 13 years** | Ref. | Ref. |  | Ref. | Ref. |  | Ref. | Ref. |
| **SII** | **Income** | **Unadjusted** | 0.16 | 0.11–0.21 |  | 0.18 | 0.14–0.22 |  | 0.17 | 0.14–0.21 |
|  |  | **Adjusted** | 0.16 | 0.11–0.21 |  | 0.18 | 0.13–0.23 |  | 0.18 | 0.14–0.21 |
|  | **Education** | **Unadjusted** | 0.13 | 0.08–0.19 |  | 0.11 | 0.05–0.16 |  | 0.14 | 0.10–0.18 |
|  |  | **Adjusted** | 0.13 | 0.08–0.19 |  | 0.11 | 0.06–0.16 |  | 0.14 | 0.10–0.18 |
| **RII** | **Income** | **Unadjusted** | 1.27 | 1.17–1.37 |  | 1.31 | 1.22–1.40 |  | 1.30 | 1.23–1.37 |
|  |  | **Adjusted** | 1.27 | 1.17–1.37 |  | 1.31 | 1.22–1.40 |  | 1.31 | 1.24–1.38 |
|  | **Education** | **Unadjusted** | 1.22 | 1.12–1.33 |  | 1.17 | 1.08–1.26 |  | 1.24 | 1.16–1.32 |
|  |  | **Adjusted** | 1.22 | 1.12–1.33 |  | 1.17 | 1.08–1.26 |  | 1.24 | 1.16–1.32 |
